# A modern approach to identifying and characterizing child asthma and wheeze phenotypes based on clinical data

**DOI:** 10.1371/journal.pone.0227091

**Published:** 2019-12-30

**Authors:** Bronwyn K. Brew, Flaminia Chiesa, Cecilia Lundholm, Anne Örtqvist, Catarina Almqvist

**Affiliations:** 1 Department of Medical Epidemiology and Biostatistics, Karolinska Institutet, Stockholm, Sweden; 2 National Perinatal Epidemiology and Statistics Unit, Centre for Big Data Research in Health and the School of Women and Children’s Health, University of New South Wales, Sydney, Australia; 3 IQVIA Nordics, Stockholm, Sweden; 4 Clinical Epidemiology Division, Department of Medicine, Karolinska Institutet, Stockholm, Sweden; 5 Department of Obstetrics and Gynecology, Visby Lasarett, Gotland, Sweden; 6 Pediatric Allergy and Pulmonology Unit, Karolinska University Hospital, Stockholm, Sweden; Telethon Institute for Child Health Research, AUSTRALIA

## Abstract

‘Asthma’ is a complex disease that encapsulates a heterogeneous group of phenotypes and endotypes. Research to understand these phenotypes has previously been based on longitudinal wheeze patterns or hypothesis-driven observational criteria. The aim of this study was to use data-driven machine learning to identify asthma and wheeze phenotypes in children based on symptom and symptom history data, and, to further characterize these phenotypes. The study population included an asthma-rich population of twins in Sweden aged 9–15 years (n = 752). Latent class analysis using current and historical clinical symptom data generated asthma and wheeze phenotypes. Characterization was then performed with regression analyses using diagnostic data: lung function and immunological biomarkers, parent-reported medication use and risk-factors. The latent class analysis identified four asthma/wheeze phenotypes: *early transient wheeze* (15%); *current wheeze/asthma* (5%); *mild asthma* (9%), *moderate asthma* (10%) and a healthy phenotype (61%). All wheeze and asthma phenotypes were associated with reduced lung function and risk of hayfever compared to healthy. Children with mild and moderate asthma phenotypes were also more likely to have eczema, allergic sensitization and a family history of asthma. Furthermore, those with moderate asthma phenotype had a higher eosinophil concentration (β 0.21, 95%CI 0.12, 0.30) compared to healthy and used short-term relievers at a higher rate than children with mild asthma phenotype (RR 2.4, 95%CI 1.2–4.9). In conclusion, using a data driven approach we identified four wheeze/asthma phenotypes which were validated with further characterization as unique from one another and which can be adapted for use by the clinician or researcher.

## Introduction

Asthma is a heterogeneous disease often characterized by wheeze, cough, chest tightness and shortness of breath caused by multiple triggers, and changes over the life course [1]. There has been a recent focus on disentangling the heterogeneity in order to identify specific phenotypes and endotypes for the purposes of better management and treatment of asthma and wheezing illnesses [2–8]. A number of modern data-driven machine learning approaches have been used to identify phenotypes such as latent class analysis (LCA) [9, 10]. The data-driven approach is hypothesis-free relying on the statistical model to generate clusters of phenotypes based on the variables added to the model rather than pre-formulated hypotheses, and has been shown to be appropriate for use in complex diseases such as asthma [9].

To date, the variables used for LCA analysis in children have consisted of wheeze patterns [6, 8, 11], growth patterns [12], atopic status [13–15] or a range of diagnostic criteria [10, 16, 17]. However, the majority of these studies are based on detailed longitudinal information from selected cohorts that while useful in understanding disease progression, can be difficult to generalize to the average patient seen in the clinic on an irregular basis. Therefore, it is of value to focus on wheeze and asthma symptoms as well as symptom history that would be typically used in a clinician-led history, or in a questionnaire by researchers. The aim of this study was to first use data driven approach to identify asthma and wheeze phenotypes based on symptom history data and secondly to confirm that these phenotypes were relevant for clinicians and researchers by further characterization using diagnostic tests, biomarkers, asthma medication and risk factor history information.

## Methods

### Study population

The Childhood and Adolescent Twin Study in Sweden cohort (CATSS) study is a continually recruiting cohort that recruits all 9 and 12 year old twins born in Sweden from July 1992 onwards for participation in interviews on health and development [18]. The Swedish Twin study on Prediction and Prevention of Asthma (STOPPA) cohort is an asthma rich cohort recruited from the CATSS.[19] STOPPA has been described and reported on previously [19]. In brief, the goal of the STOPPA cohort was to identify an asthma rich cohort from CATSS that could be studied in more depth with clinical and biometric examination. Based on questions validated through the International Study of Asthma and Allergies in Childhood (ISAAC) [20] in the CATSS interview material at age 9 or 12, an algorithm was created to identify same sex twins born 1997–2004 who: both had asthma (concordant asthma), one had asthma and the other did not (discordant), or both had no asthma (healthy concordant). In total, 6,174 twins were eligible for STOPPA, however, since discordants only made up 13% of all eligible twins and the objective was to recruit equal numbers of twin pairs with concordant asthma, concordant healthy and discordant, a sample of 1,448 were contacted, 870 agreed to participate and 752 came to the clinical examination, a response rate of 52%.

The STOPPA cohort participated in clinical testing and their parents completed questionnaires when the children were 9–15 years of age. Data in STOPPA was linked by personal identity number to nation-wide registers held in Sweden by the National Board of Health and Welfare and Statistics Sweden, including the Medical Birth Register (MBR) and the Longitudinal Integration Database for health insurance and labour market studies (LISA) [21].

The study was approved by the Regional Ethical review board in Stockholm, Sweden. Informed written consent for the study was obtained from all children and their parents.

### Variables used in the latent class analysis

The 17 variables used for the LCA were based on wheeze and asthma symptoms. The justification for these variables was that they are representative of the questions a clinician may ask patients and their parents when taking a ‘patient history’ in order to determine an asthma phenotype and subsequent disease management. The symptom and symptom history variables were based on ISAAC questions [20] and the Global Initiative for Asthma (GINA) guidelines [1] (S1 Table). *Ever asthma*, *age of FIRST asthma*, *wheezing or breathlessness attack* and *age of LAST breathlessness attack* were reported by parents during the initial CATSS interview when the twins were 9 years old. The other asthma and wheeze questions were parent-reported at STOPPA recruitment. These included: *ever wheeze*, *wheezing episode in the last 12 months*- if ‘yes’, *how many times*?, *wheeze due to a cold*, *current snoring* and *current asthma*. If *current asthma* was ‘yes’ then the following variables were also asked about: *asthma diagnosed by a doctor* and the *age of asthma diagnosis*; and in the last 12 months- *limited speech to one/two words per breath due to asthma; breathing difficulties due to asthma*; *woken by asthma*; *disturbed in daily activity due to asthma; acute visit to emergency or general practitioner for asthma; admitted to hospital for asthma*. For all who answered ‘no’ to *current asthma*, these variables were all set to ‘no’.

### Variables used to further characterize asthma and wheeze phenotypes

#### Possible risk factors

*Parental history of asthma*, *environmental tobacco smoking exposure*, *breast-feeding exposure* and *history of dog ownership* were parent-reported in STOPPA. *Birth weight*, *gestational age* and *parity* were obtained from the MBR, and *highest parental education gained* from the LISA.

#### Allergy and immunological variables

The child *ever having eczema* and *ever having hayfever* were parent-reported. *Allergic sensitivity* was recorded as positive if sera measured ≥ 0.35kU/l for Phadiatop^®^ (Thermo Fisher Scientific, Uppsala, Sweden). Those positive were then further analysed for specific IgE (sIgE) antibodies to the single allergens of *cat*, *dog*, *horse* and *birch*. A sIgE result ≥ 0.7kU/l was considered to be positive. Those participants who were negative for Phadiatop^®^ were assigned the value 0.09 kU/l (the level below quantification) for each sIgE.[22] A complete blood count of blood samples provided information on *eosinophil* and *neutrophil granulocyte concentrations* (cells x10^9^/L) as well as *lymphocyte particle concentrations* (cells x10^9^/L).

#### Asthma medication

History of the child’s asthma medication was parent-reported in STOPPA. Parents were initially asked if the *child currently took any asthma medication*. If they answered ‘yes’, they were then asked if the child had taken a *short-acting medication (β2 agonist) in the last week*, and details about whether the child took regular or periodic asthma medication in the last 12 months including: *inhaled corticosteroids (ICS)*, *long-acting β2 agonists*, *combination medications of ICS and fast-acting β2 agonists*, *leukotriene receptor antagonists (LTRA)* or *systemic corticosteroids*.

#### Lung function and fractional exhaled NO (FeNO)

*FeNO* (parts per billion), a noninvasive biomarker of airway inflammation, was measured with a hand-held electrochemical analyzer (NIOXMino, Aerocrine, Solna, Sweden) or FeNO test analyzer (Ecomedics, Duernten, Switzerland). Each subject performed the test at least twice, if there was *>*5% difference between the first two measurements a third attempt was performed [19].

Participants performed spirometry [19] to ascertain forced expiratory volume in the first second after a maximal inhalation (FEV1) and forced vital capacity- total volume expired after maximal inhalation (FVC) before and after 15 minutes of inhaling a bronchodilator (0.5mg of terbutalin) to test reversibility. At least three attempts with high reproducibility (<0.15L between two highest values) were required for each procedure and the maximum value of the attempts was used for the analyses. Lung function values were converted to z-scores based on the Global Lung Function Initiative reference values, taking sex, age, height and ethnicity into account.[23] Reversibility was calculated as a percentage change in FEV1 from baseline: (postfev1-prefev1) /prefev1*100.

### Statistical analysis

The LCA was conducted in MPLUS version 7.31 (Muthen & Muthen, Los Angeles, CA) to determine phenotypes of wheeze and asthma based on the unsupervised associations found between variables. Individuals were assigned to the class for which they had the highest posterior probability of belonging. Starting with a model assuming 2 phenotypes we compared model fit of increasing numbers of phenotypes (up to 7) using the Bayesian information criteria (BIC), Aikake information criteria (AIC) and the Lo Mendell Rubin test (LMR). Entropy index was used to determine the goodness of fit of the data to the number of classes. Different starting values for the algorithm iterations were used to avoid local maxima. Results in the LCA are presented as conditional probabilities (CP). These represent the probability of an individual in a given class of the latent variable being at a particular level of the observed variable. Based on CP within each latent class, a label for each class was determined by the authors.

In order to characterize the phenotypes further, proportions and means of potential risk factors, allergy, immunological markers, medication use, FeNO and lung function were calculated. Supervised analysis involved generalized linear models to calculate relative risks (RR) for dichotomous variables and β-estimates for continous variables comparing each latent class with the ‘healthy’ phenotype. Only significant associations are presented. For allergy, immunological markers, asthma medication and respiratory function comparisons with RR, β-estimates and 95% CI were also made between the ‘moderate asthma’ phenotype and the ‘mild asthma’ phenotype to provide further differentiation between these two asthma phenotypes. We accounted for clustering of observations within twin pairs by using the robust sandwich estimator for standard errors. Any missing data was assumed to be missing at random.

Data management and statistical analyses (apart from the LCA) were conducted using SAS 9.4 (SAS Institute Inc., USA) and STATA 15.1 (StataCorp, USA).

## Results

The best fitting LCA was the 5 class model based on the lowest BIC and AIC, the LMR which suggested that this model was significantly different to the 4 class model, and the entropy index approaching 1 (S2 Table). Table 1 displays the conditional probabilities of each symptom or symptom history that were included in the LCA for each latent class. The five phenotypes were given labels to best describe the profile of conditional probabilities: ‘Healthy’, ‘Early transient wheeze’, ‘Current wheeze/asthma’, ‘Mild asthma’ and Moderate asthma’. The class probability, i.e. the probability of belonging to the class the individual was assigned to, was higher than 0.5 for all individuals.

**Table 1:** Conditional probabilities of symptom and symptoms history for each class of the 5 class model, STOPPA cohort, age 9–15 years, N = 752.

	Number of cases	Healthy N = 459	Early transient wheeze N = 114	Current wheeze/Asthma N = 38	Mild asthma N = 66	Moderate Asthma N = 75
**Ever wheeze**	324	0.14	0.82	0.97	0.74	1.00
**Wheezing episode in the last 12 months**	113	0	0	1.00	0	1.00
**-1-3 times**	65	0	0	0.76	0	0.48
**-≥ 4 times**	47	0	0	0.21	0	0.52
**-due to a cold**	84	0	0	0.50	0	0.87
**Ever asthma**	233	0.02	0.79	0.32	0.80	0.85
**age of** **first** **asthma, wheezing or breathlessness attack ≤ 2 years**	260	0.61	0.85	0.77	0.73	0.57
**>2 years**	110	0.39	0.15	0.23	0.27	0.43
**-age of** **last** **asthma, wheezing or breathlessness attack ≤ 4 years**	76	0.17	0.59	0.40	0.42	0
**>4 years**	143	0.83	0.41	0.60	0.58	1.00
**Current asthma**	140	0	0	0	1.00	1.00
**-asthma diagnosis**	135	0	0	0	0.99	0.95
**-age of asthma diagnosis ≤2yrs**	11	0	0	0	0.17	0.09
**>2yrs**	78	0	0	0	0.83	0.92
**-asthma has limited speech to 1/2 words in between asthma attacks in the last 12 months**	5	0	0	0	0	0.07
**-breathing difficulties due to asthma >once/week in the last 12 months**	78	0	0	0	0.30	0.77
**-awakened by asthma** **>once/month in the last 12 months**	8	0	0	0	0.03	0.08
**-disturbed in daily activities due to asthma in the last 12 months**	85	0	0	0	0.42	0.76
**-visited doctor for asthma in the last 12 months**	8	0	0	0	0	0.11
**-admitted to hospital due to asthma in the last 12 months**	2	0	0	0	0	0.03
**Current snoring**	97	0.12	0.16	0.19	0.14	0.15

### Early transient wheeze

**Fig 1:**
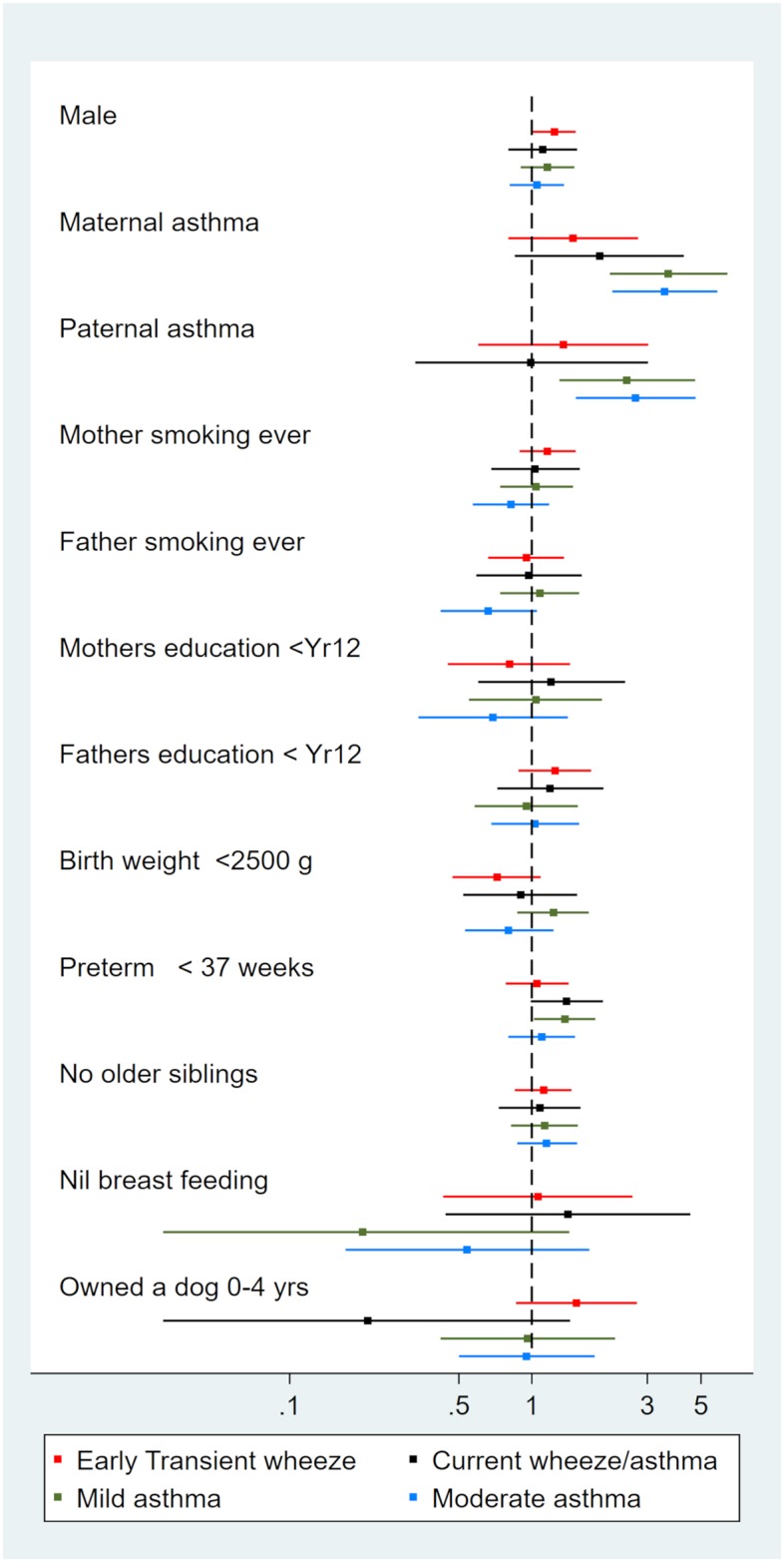
Risk factors for latent class phenotypes compared to healthy phenotype. Relative risks and 95% confidence intervals.

**Fig 2:**
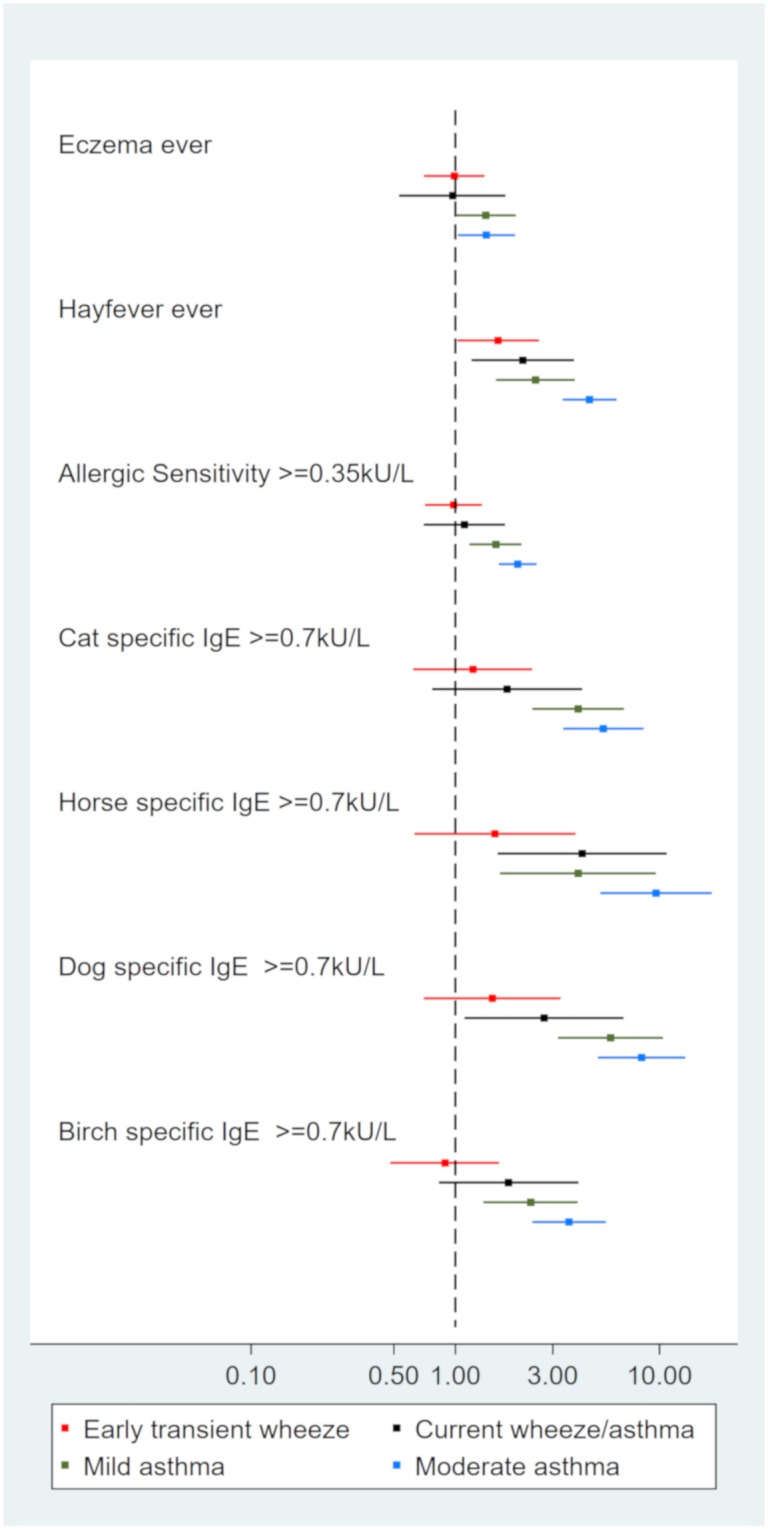
Allergy profile for latent class phenotypes compared to healthy phenotype. Relative risks and 95% confidence intervals.

Children with this phenotype did not report current wheeze or asthma (Table 1). However they had a history of wheeze or asthma (CP 82% ever wheeze and 79% ever asthma), most of the asthma cases beginning before two years of age. Characterization: children with ‘early transient wheeze’ phenotype did not differ in risk factors to the ‘healthy’ phenotype, although 61% of this group were boys (p = 0.05), Fig 1 and Table 2. They had a similar allergy and immune profile to the ‘healthy’ phenotype (Table 3 and Fig 2), and were unlikely to use any asthma medication (Table 4). The most outstanding feature of this phenotype was despite having no reported current asthma or wheeze the children were more likely to have reduced lung function than ‘healthy’ children having lower pre and post FEV1. They also had a greater degree of reversibility, 4.87 ±5.14%, p<0.01 (Table 5).

**Table 2:** Risk factors for latent class phenotypes.

	Healthy n (%)	Current wheeze/asthma n (%)	Early transient wheeze n (%)	Mild asthma n (%)	Moderate asthma n (%)
**Male**	228 (49.7)	21 (55.3) RR 1.1 (0.8, 1.5)	**70 (61.4)** **RR 1.2 (1.0, 1.5)**	38 (57.6) RR 1.2 (0.9, 1.5)	39 (52.0) RR 1.1 (0.8, 1.4)
**Maternal asthma**	38 (8.3)	6 (15.8) RR 1.9 (0.9, 4.3)	14 (12.3) 1.5 (0.8, 2.8)	**20 (30.3)** **RR 3.7 (2.3, 5.9)** ^c^	**22 (29.3)** **RR 3.5 (2.2, 5.6)**^c^
**Paternal asthma**	36 (7.8)	3 (7.9) RR 1.0 (0.3, 3.0)	12 (10.5) RR 1.4 (0.6, 3.0)	**13 (19.7)** **RR 2.7 (1.5, 4.8)**^b^	**16 (21.3)** **RR 3.1 (1.9, 5.3)**^c^
**Mother smoking ever**	187 (40.7)	16 (42.1) RR 1.0 (0.7, 1.6)	54 (47.4) RR 1.2 (0.9, 1.5)	28 (42.4) RR 1.0 (0.7, 1.5)	25 (33.3) RR 0.8 (0.6, 1.2)
**Father smoking ever**	159 (35.1)	13 (34.2) RR 1.0 (0.6, 1.6)	36 (33.3) RR 1.0 (0.7, 1.4)	25 (37.9) RR 1.1 (0.7, 1.6)	17 (23.3) RR 0.7 (0.4, 1.1)
**Mothers education < year 12**	79 (17.5)	8 (21.1) RR 1.2 (0.6, 2.4)	16 (14.2) RR 0.8 (0.5, 1.4)	12 (18.2) RR 1.0 (0.6, 2.0)	9 (12.2) RR 0.7 (0.3, 1.4)
**Fathers education < year 12**	130 (28.8)	13 (34.2) RR 1.2 (0.7, 2.0)	41 (36.0) RR 1.3 (0.9, 1.8)	18 (27.3) RR 1.0 (0.6, 1.6)	22 (29.7) RR 1.0 (0.7, 1.6)
**Birth weight underweight <2500 g**	138 (37.1)	10 (33.3) RR 0.9 (0.5, 1.5)	24 (26.7) RR 0.7 (0.5, 1.1)	25 (45.5) RR 1.2 (0.9, 1.7)	20 (29.9) RR 0.8 (0.5, 1.2)
**Preterm < 37 weeks**	168 (38.8)	20 (54.1) RR 1.4 (1.0, 2.0)	46 (40.7) RR 1.1 (0.8, 1.4)	**34 (53.1)** **RR 1.4 (1.1, 1.8)**^a^	32 (42.7) RR 1.1 (0.8, 1.5)
**No older siblings**	191 (41.6)	17 (44.7) RR 1.1 (0.7, 1.6)	53 (46.5) RR 1.1 (0.9, 1.5)	31 (47.0) RR 1.1 (0.8, 1.6)	36 (48.0) R 1.2 (0.9, 1.5)
**Nil breast feeding**	34 (7.4)	4 (10.5) RR 1.4 (0.4, 4.5)	9 (7.9) RR 1.1 (0.4, 2.6)	1 (1.5) RR 0.2 (0.03, 1.4)	3 (4.0) RR 0.5 (0.2, 1.7)
**Owned a dog 0–4 yrs**	58 (12.6)	1 (2.6) RR 0.2 (0.0, 1.4)	22 (19.3) RR 1.5 (0.9, 2.7)	8 (12.1) RR 1.0 (0.4, 2.2)	9 (12.0) R 1.0 (0.5, 1.8)
**Age at examination** **(mean ± sd)**	12.64±1.42	12.45±1.41	12.30±1.42	12.26±1.52	12.43±1.58

^a^ p <0.05,

^b^ p< 0.001,

^c^ p<0.0001

Proportion per latent class group and Relative risks (RR) with 95% confidence intervals compared to healthy phenotype. N = 752.

**Table 3:** Immunological profile for latent class phenotypes.

	Healthy mean ± SD	Early transient wheeze mean ± SD	Current wheeze/asthma mean ± SD	Mild asthma mean ± SD	Moderate asthma mean ± SD
**Eosinophil granulocyte conc (cells x10**^**9**^**/l)**	0.25 ±0.23	0.30±0.36 β 0.04 (-0.03, 0.12)	0.25±0.21 β 0.00 (-0.08, 0.08)	0.33±0.30 β 0.08 (0.00, 0.16)	**0.46±0.38** **β 0.21 (0.12, 0.30)**^b^
**Lymphocyte particle conc (cells x10**^**9**^**/l)**	2.50 ±0.63	2.56±0.63 β 0.06 (-0.08, 0.19)	2.37±0.72 β -0.13 (-0.39, 0.12)	**2.78±0.75** **β 0.27 (0.06, 0.49)**^a^	2.65±0.76 β 0.15 (-0.05, 0.35)
**Neutrophil granulocyte conc (cells x10**^**9**^**/l)**	3.17±1.29	3.11±0.99 β -0.06 (-0.30, 0.17)	3.29±1.71 β 0.12 (-0.57, 0.81)	3.09±1.15 β -0.08 (-0.43, 0.26)	2.99±0.91 β -0.19 (-0.45, 0.07)

^a^ p <0.05,

^b^ p<0.0001

Beta coefficients with 95% confidence intervals compared to healthy phenotype.

**Table 4:** Asthma medication use for latent class phenotypes.

	Early transient wheeze n (%)	Current wheeze/ asthma n (%)	Mild asthma n (%)	Moderate asthma n (%)
**Any current asthma medication**	4 (3.9)	4 (11.1)	37 (56.1)	**61 (81.3)** **RR 1.5 (1.1, 1.9)**^b^
**β2 short- acting medication more than twice in the last week**	2 (2.0)	2 (5.6)	10 (15.2)	**26 (35.6)** **RR 2.4 (1.2, 4.9)**^a^
**Any *regular* preventer medication in the last 12 months**	0 (0)	0 (0)	17 (28.8)	**29 (40.9)** **RR 1.6 (1.0, 2.5)**^a^
**Any *periodic* preventer medication in the last 12 months**	3 (3.0)	5 (13.9)	21 (36.8)	**38 (58.5)** **RR 1.7 (1.1, 2.5)**^b^
***Regular* ICS medication in the last 12 months**	0 (0)	0 (0)	9 (15.8)	13 (20.6) 1.8 (0.8, 3.7)
***Periodic* ICS medication in the last 12 months**	1 (1.0)	4 (11.4)	17 (29.8)	**30 (47.6)** **RR 1.7 (1.1, 2.7)**^a^
***Regular* β2 ICS combination medication in the last 12 months**	0 (0)	0 (0)	11 (19.3)	19 (28.8) RR 1.6 (0.8, 3.3)
***Periodic* β2 ICS combination medication in the last 12 months**	2 (2.0)	2 (5.7)	5 (8.8)	8 (12.1) RR 1.6 (0.6, 4.4)
***Regular* β2 long-acting medication in the last 12 months**	0 (0)	0 (0)	2 (3.6)	5 (8.6) RR 2.5 (0.6, 9.9)
***Periodic* β2 long-acting medication in the last 12 months**	2 (2.0)	1 (2.9)	7 (12.7)	9 (15.5) RR 1.3 (0.5, 3.3)
***Regular* LTRA medication in the last 12 months**	0 (0)	0 (0)	6 (10.5)	5 (8.2) RR 0.8 (0.2, 2.7)
***Periodic* LTRA medication in the last 12 months**	1 (1.0)	0 (0)	5 (8.8)	4 (6.6) RR 0.7 (0.2, 3.3)
**Systemic corticosteroid in the last 12 months**	1 (1.0)	3 (8.8)	3 (5.4)	3 (5.2) R 1.0 (0.2, 4.6)

^a^ p <0.05,

^b^ p< 0.001,

Relative risks (RR) and 95% confidence intervals shown for Moderate asthma versus Mild asthma.

**Table 5:** Respiratory function of latent class phenotypes.

	Healthy mean ± SD	Early transient wheeze mean ± SD	Current wheeze/asthma mean ± SD	Mild asthma mean ± SD	Moderate asthma mean ± SD
**FENO (parts per billion) n = 705**	16.04± 13.61	15.73± 14.18 β -0.31 (-3.47, 2.86)	15.76± 17.57 β -0.28 (-6.21, 5.65)	**22.09± 20.77** **β 6.05 (0.88, 11.22)**^a^	**27.18± 22.21** **β 11.14 (5.52, 16.77)**^c^
**PreFEV1 z score** **n = 576**	-0.40± 0.97	**-0.76± 0.91** **β-0.36 (-0.61, -0.12)**^b^	-0.67± 1.11 β -0.27 (-0.69, 0.15)	**-0.72± 0.89** **β -0.32 (-0.62, -0.02)**^a^	**-0.78± 1.04** **β-0.38 (-0.68, -0.08)**^a^
**PreFVC z score** **n = 576**	-0.26± 0.99	-0.39± 0.88 β -0.13 (-0.36, 0.11)	-0.26± 0.92 β 0.01 (-0.35, 0.36)	-0.12± 0.91 β 0.15 (-0.17, 0.46)	-0.26± 0.97 β 0.01 (-0.27, 0.28)
**PreFEV1FVC z score ratio** **n = 576**	-0.23± 1.02	**-0.62± 1.10** **β -0.39 (-0.69, -0.09)**^a^	**-0.70± 1.08** **β -0.47 (-0.86, -0.07)**^a^	**-0.96± 1.05** **β -0.72 (-1.08, -0.037)**^c^	**-0.81± 1.15** **β -0.58 (-0.92, -0.24)**^b^
**PostFEV1 z score** **n = 588**	-0.18± 0.97	**-0.46± 0.86** **β -0.29 (-0.53, -0.06)**^a^	-0.31± 0.98 β -0.14 (-0.50, 0.22)	-0.27± 0.89 β -0.10 (-0.38, 0.18)	-0.40± 1.04 β -0.23 (-0.52, 0.07)
**PostFVC z score** **n = 588**	-0.24± 0.91	-0.36± 0.84 β -0.11 (-0.33, 0.11)	-0.29± 0.90 β -0.04 (-0.37, 0.28)	-0.18± 0.88 β 0.06 (-0.23, 0.35)	-0.28± 1.04 β -0.03 (-0.32, 0.25)
**PostFEV1FVC z score ratio** **n = 588**	0.10± 0.97	**-0.17±1.07** **β -0.28 (-0.55, 0.00)**^a^	-0.07± 1.14 β -0.17 (-0.58, 0.25)	**-0.18±0.94** **β -0.28 (-0.56, 0.00)**^a^	-0.18±1.05 β -0.28 (-0.58, 0.02)
**Reversibility (%)** **n = 478**	2.58± 5.01	**4.87± 5.14** **β 2.29 (0.89, 3.69)**^b^	4.73± 5.77 β 2.14 (-0.11, 4.40)	**4.09± 3.95** **β 1.51 (0.16, 2.85)**^a^	**5.36± 6.78** **β 2.77 (0.75, 4.80)**^b^

^a^ p <0.05,

^b^ p< 0.001,

^c^ p<0.0001,

**some missing data due to not all completing spirometry

Beta coefficients and 95% confidence intervals compared to healthy phenotypes. N = 705**

### Current wheeze/asthma

Children with this phenotype typically had wheeze in the last 12 months (Table 1). Half attributed their wheeze to having a cold, some reported ever asthma (CP 32%) but not current asthma. Characterisation: these children did not differ in risk factors to the ‘healthy’ phenotype (Fig 1, Table 2). They were twice as likely to have hayfever and had an increased risk of horse and dog specific allergy compared to ‘healthy’ phenotype (Table 3, Fig 2). In addition, these children had a slightly lower lung function than the ‘healthy‘ phenotype (Table 5) and minimal asthma medication use (Table 4). Taken together this phenotype seems to represent wheeze due to allergy or viruses or mild undiagnosed asthma.

### Mild asthma

Children with this phenotype reported current asthma but no wheeze in the last 12 months (Table 1). However, some children reported symptoms due to asthma including; breathing difficulties and disturbance in daily activities. Characterisation: children with ‘mild asthma’ were more likely to have a family history of asthma, and more likely to be born preterm than the ‘healthy’ phenotype (Fig 1, Table 2). These children had a notable allergic profile. They were more likely to have ever eczema, hayfever and allergic sensitivity compared to ‘healthy’ and had IgE sensitivity to each of cat, dog, horse and birch (Fig 2, Table 6). In addition they were more likely to have a heightened lymphocyte response (Table 3). 56% of children with ‘mild asthma’ phenotype used asthma medication in the last 12 months. This included 29% using regular preventers and 37% using periodic preventers (Table 4). Only 15% had taken a short acting beta-agonist (SABA) more than twice in the last week. Average exhaled NO concentration in these children was higher than ‘healthy’ by 6.05 parts per billion (Table 5). PreFEV1 and pre FEV1FVC ratio were reduced, but improved to ‘healthy’ phenotype lung function after taking terbutaline (Table 5).

**Table 6:** Allergy profile for latent class phenotypes.

	Healthy n (%)	Current wheeze/asthma n (%)	Early transient wheeze n (%)	Mild asthma n (%)	Moderate asthma n (%)
**Eczema ever**	132 (28.8)	10 (27.0) RR 1.0 (0.5, 1.8)	33 (28.9) RR 1.0 (0.7, 1.4)	**27 (40.9)** **RR 1.4 (1.0, 2.0)** ^a^	**31 (41.9)** **RR 1.4 (1.0, 2.0)** ^a^
**Hayfever ever**	62 (13.5)	**11 (28.9)** **RR 2.1 (1.2, 3.8)**^a^	**25 (21.9)** **RR 1.6 (1.0, 2.6)** ^a^	**22 (33.3)** **RR 2.5 (1.6, 3.8)**^c^	**46 (61.3)** **RR 4.5 (3.3, 6.2)** ^c^
**Allergic sensitivity (phadiatop©≥35ku/l)**	147 (32.0)	13 (34.2) RR 1.1 (0.7, 1.8)	37 (32.5) RR 1.0 (0.7, 1.4)	**32 (48.5)** **RR 1.6 (1.2, 2.1)** ^b^	**48 (64.0)** **RR 2.0 (1.6, 2.5)** ^c^
**Cat specific IgE (>0.7ku/l)**	35 (7.6)	5 (13.2) RR 1.8 (0.8, 4.2)	11 (9.7) RR 1.2 (0.6, 2.4)	**19 (28.8)** **RR 4.0 (2.4, 6.7)** ^c^	**30 (40.0)** **RR 5.3 (3.4, 8.4)** ^c^
**Horse specific IgE (>0.7ku/l)**	15 (3.3)	**5 (13.2)** **RR 4.2 (1.6, 10.9)**^b^	6 (5.3) RR 1.6 (0.6, 3.9)	**8 (12.1)** **RR 4.0 (1.7, 9.6)** ^b^	**23 (30.7)** **RR 9.6 (5.1, 18.0)** ^c^
**Dog specific IgE (>0.7ku/l)**	23 (5.0)	**5 (13.2)** **RR 2.7 (1.1, 6.7)**^b^	9 (7.9) RR 1.5 (0.7, 3.3)	**18 (27.3)** **RR 5.7 (3.2, 10.4)** ^c^	**30 (40.0)** **RR 8.2 (5.0, 13.4)** ^c^
**Birch specific IgE (>0.7ku/l)**	48 (10.5)	7 (18.4) RR 1.8 (0.8, 4.0)	11 (9.7) RR 0.9 (0.5, 1.6)	**15 (22.7)** **RR 2.3 (1.4, 4.0)** ^b^	**28 (37.3)** **RR 3.6 (2.4, 4.5)** ^c^

^a^ p <0.05,

^b^ p< 0.001,

^c^ p<0.0001

Proportion per latent class group and Relative risks (RR) with 95% confidence intervals compared to healthy. N = 752

### Moderate asthma

Children with this phenotype had current asthma and wheeze that was disturbing their lives. Approximately half had experienced more than four episodes of wheeze in the last twelve months (Table 1). All reports of uncontrolled asthma symptoms in the last 12 months were found in this cluster–asthma limiting speech to one or two words between asthma attacks (n = 5), admission to hospital (n = 2), unscheduled visit to the emergency or general practitioner for asthma (n = 8). In addition, there was a high probability of having breathing difficulties due to asthma more than once per week, or disturbance to daily activities by asthma in the last 12 months. Characterisation: similar to ‘mild asthma’, children with ‘moderate asthma’ phenotype were more likely to have a family history of asthma compared to ‘healthy’ phenotype (Fig 1, Table 2). A notable feature of this group was the risk of also having hayfever was higher than both the Table 6). This pattern was similar for allergic sensitivity as well as for each of the specific allergens and eosinophil count (Table 3).

In comparison with ‘mild asthma’ phenotype, the ‘moderate asthma’ phenotype were more likely to be using: any asthma medication (RR 1.5 95%CI 1.1, 1.9), SABA twice a week (RR 2.4, 95%CI 1.2, 4.9), and regular (RR 1.6, 95%CI 1.0, 2.5) or periodic (RR 1.7, 95%CI 1.1, 2.5) preventers in the last 12 months (Table 4). They had higher exhaled NO concentration than ‘healthy’ by 11.14, SE 2.9 parts per billion (Table 5). The respiratory function results were decreased but not significantly different to the ‘mild asthma’ phenotype (Table 5).

## Discussion

Using hypothesis-free testing based on asthma and wheeze symptoms and symptom history we identified four clusters of disease in children and young adolescents: early transient wheeze, current wheeze/asthma, mild asthma and moderate asthma. Further characterization of these phenotypes based on risk factors, allergy and immune profiles, asthma medication use and lung function testing in comparison to a healthy group reinforced the uniqueness of each of these disease states which can be used to better understand and manage asthma and wheezing illness.

‘Early transient wheeze’ is a well-recognized phenotype and our results are consistent with others findings: ‘early transient wheezers’ have no or low atopy, no current wheeze, minimal asthma medication use, and stable FeNO [5, 6, 8, 11]. However, despite no current reported symptoms, lung function in this group is reduced and is more prevalent in boys [5–7, 11]. An explanation for ‘early transient wheeze’ with decreased lung function could be that these children have smaller lungs and more restricted airways in early life. The Tucson study found that children with diminished lung function at birth were more likely to have early wheeze [24] and that these same children continued to have impaired lung function at age 22 [25]. This finding that early wheeze phenotype is associated with long term impaired lung function has been confirmed by other studies [7, 26]. Taken together, this may mean that children with early wheeze may be at risk of obstructive lung disease later in life even if the wheeze is transient and they appear to have no other asthmatic or allergic symptoms.

The ‘current wheeze/asthma’ phenotype appears to be a generally healthy cluster with occasional wheeze triggered by a range of sources such as viruses and/or allergens. However, it may be useful to identify wheeze triggers and avoidance strategies within this group. A similar phenotype in slightly older adolescents was identified in the Isle of Wight study as ‘undiagnosed wheezers’ with strong associations with paracetamol use and smoking [27].

Our study found two phenotypes of current asthma identified as ‘mild asthma’ and ‘moderate asthma’ in the LCA. The different labels were based on the effect of asthma on day to day life in terms of: disturbing daily activity, waking up from asthma, exacerbations and recent wheeze, all of which were higher in the disturbing asthma phenotype. Both these asthma phenotypes have similarities with ‘persistent’ wheeze found in other longitudinal wheeze studies, including allergic sensitization, reduced lung function, increased reversibility, parental asthma, and higher FeNO [5, 8, 17]. It may be therefore that we have identified two phenotypes within the persistent wheeze phenotype. Belgrave et al. used a longitudinal latent class item response model to identify clusters based on parental and clinician reported wheeze over 8 years. They also were able to split persistent wheeze into two further groups ‘persistent controlled wheeze’ and ‘persistent troublesome wheeze’ [11]. Those with persistent troublesome wheeze have a similar profile to our ‘moderate asthma’, that is, they were more likely to be sensitized, have eczema, use ICS medication, have hospital admissions and asthma exacerbations compared to persistent controlled wheezers. A concerning issue is that a large proportion of the children with ‘moderate’ asthma identified in the STOPPA cohort have regular wheeze and asthma-related disturbances, and use reliever medication regularly suggesting the asthma is not well controlled. However, less than half take regular medication for their asthma, most using their preventer medication periodically. Taken together, this would suggest that the health and quality of life of those with moderate asthma phenotype would benefit from better medication adherence and monitoring of disease.

This is the first study that we know of that has applied latent class analysis to asthma and wheezing symptoms and symptom history taken at a single point in time rather than longitudinally. Although longitudinal analysis is superior in many ways to the cross-sectional study, we sought to identify and characterize phenotypes based on a series of questions that can be used either by a clinician taking a patient history, or for a researcher conducting a survey. The question included in the LCA variables were based on the asthma and wheeze questions in ISAAC [20] and GINA [1] and follow questions commonly asked by clinicians during a consultation with a child and their parent. The value of using LCA is that the cluster groups are not chosen *a priori* but are determined statistically based on the assumption that all associations between the included variables are due to unobserved latent classes representing disease-specific mechanisms or endotypes, that is, the analysis is ‘unsupervised’ [10]. The variables used in the characterization or ‘supervised’ analysis align with further follow up questions regarding risk factors, allergies, medication use and diagnostic tests that the clinician may apply. These confirmed that the phenotypes the LCA had revealed are unique, have clinical relevance and overlap with those found in other studies.

In regards to limitations, this study was cross-sectional which although makes it relevant to apply to patients seen in an irregular manner in clinics it does not capture disease trajectory, nor is it possible to assess the predictive validity of the observed classes for any outcomes. Secondly, there may be bias in the LCA due to parent recall—over or under reporting of the variables, or because of symptom modification from medication usage. Another possible limitation with our study is that it is not as large as other studies and may lack further breakdown into even more specific clusters. However, as the STOPPA cohort is an asthma-rich cohort with asthma pairs selected for inclusion we had increased power to discover asthma and wheeze clusters. Finally, there may be issues with generalizability to singletons as twins which are generally are born earlier and smaller (as can be seen in Table 2). However, the risk of asthma conferred by smaller birth weight and gestational age is observed in young twins, and has dissipated by older childhood on which our study is based.[28]

In conclusion, unsupervised analysis of data from respiratory symptom and symptom history questions identified four wheeze/asthma phenotypes and one healthy phenotype in children and adolescents that were shown to have unique physiological, immunological and medication profiles. These phenotypes are largely similar to others found in literature based on longitudinal data, therefore supporting the validity of symptom and symptom history data as a means of identifying clinical and research relevant phenotypes. Further characterization of these phenotypes highlighted that children and adolescents with moderate asthma may be underutilizing preventer medication and over-utilizing acute medications, therefore, reinforcing the continued need for monitoring and treatment management of children with moderate asthma whose asthma.

## Supporting information

S1 Table Questions from CATSS and STOPPA questionnaires. (DOCX)

S2 Table Comparison of 4, 5 and 6 latent class models. AIC = Aikake Information Criteria, BIC = Bayesian Information Criteria.
(DOCX)

## Abbreviations

AIC: Aikake Information Criteria
BIC: Bayesian Information Criteria
CATSS: Childhood and Adolescent Twin Study of Sweden
CI: Confidence Interval
CP: Conditional Probability
FeNO: Fractional exhaled nitric oxide
FEV1: forced expiratory volume in 1 second
FVC: forced volume capacity
GINA: Global Initiative for Asthma
ICS: inhaled corticosteroids
ISAAC: International Study of Asthma and Allergies in Childhood
LCA: Latent Class Analysis
LISA: Longitudinal Integration Database for health insurance and labour market studies
LRTA: leukotriene receptor antagonists
MBR: Medical Birth Register
RR: Relative risk
SABA: short acting beta-agonist
STOPPA: Swedish Twin study on Prediction and Prevention of Asthma

## References

[1] Global Strategy for Asthma Management and Prevention, Global Initiative for Asthma (GINA) 2016 [2017-01-22]. www.ginasthma.org.

[2] Deliu M, Belgrave D, Sperrin M, Buchan I, Custovic A. Asthma phenotypes in childhood. Expert Rev Clin Immunol. 2017;13(7):705–13. Epub 2016/11/08. 10.1080/1744666X.2017.1257940 . 27817211

[3] Deliu M, Yavuz TS, Sperrin M, Belgrave D, Sahiner UM, Sackesen C, et al Features of asthma which provide meaningful insights for understanding the disease heterogeneity. Clin Exp Allergy. 2018;48(1):39–47. Epub 2017/08/24. 10.1111/cea.13014 . 28833810 PMC5763358

[4] Hose AJ, Depner M, Illi S, Lau S, Keil T, Wahn U, et al Latent class analysis reveals clinically relevant atopy phenotypes in 2 birth cohorts. J Allergy Clin Immunol. 2017;139(6):1935–45 e12. Epub 2016/10/25. 10.1016/j.jaci.2016.08.046 . 27771325

[5] Depner M, Fuchs O, Genuneit J, Karvonen AM, Hyvarinen A, Kaulek V, et al Clinical and epidemiologic phenotypes of childhood asthma. Am J Respir Crit Care Med. 2014;189(2):129–38. Epub 2013/11/29. 10.1164/rccm.201307-1198OC . 24283801

[6] Henderson J, Granell R, Heron J, Sherriff A, Simpson A, Woodcock A, et al Associations of wheezing phenotypes in the first 6 years of life with atopy, lung function and airway responsiveness in mid-childhood. Thorax. 2008;63(11):974–80. Epub 2008/08/06. 10.1136/thx.2007.093187 . 18678704 PMC2582336

[7] Granell R, Henderson AJ, Sterne JA. Associations of wheezing phenotypes with late asthma outcomes in the Avon Longitudinal Study of Parents and Children: A population-based birth cohort. J Allergy Clin Immunol. 2016;138(4):1060–70 e11. Epub 2016/04/24. 10.1016/j.jaci.2016.01.046 . 27106203 PMC5052126

[8] Savenije OE, Granell R, Caudri D, Koppelman GH, Smit HA, Wijga A, et al Comparison of childhood wheezing phenotypes in 2 birth cohorts: ALSPAC and PIAMA. J Allergy Clin Immunol. 2011;127(6):1505–12 e14. Epub 2011/03/18. 10.1016/j.jaci.2011.02.002 . 21411131

[9] Deliu M, Sperrin M, Belgrave D, Custovic A. Identification of Asthma Subtypes Using Clustering Methodologies. Pulm Ther. 2016;2:19–41. Epub 2016/08/12. 10.1007/s41030-016-0017-z . 27512723 PMC4959136

[10] Howard R, Rattray M, Prosperi M, Custovic A. Distinguishing Asthma Phenotypes Using Machine Learning Approaches. Curr Allergy Asthma Rep. 2015;15(7):38 Epub 2015/07/06. 10.1007/s11882-015-0542-0 . 26143394 PMC4586004

[11] Belgrave DCM, Simpson A, Semic-Jusufagic A, Murray CS, Buchan I, Pickles A, et al Joint modeling of parentally reported and physician-confirmed wheeze identifies children with persistent troublesome wheezing. J Allergy Clin Immunol. 2013;132(3):575–83 e12. Epub 2013/08/03. 10.1016/j.jaci.2013.05.041 . 23906378

[12] Rzehak P, Wijga AH, Keil T, Eller E, Bindslev-Jensen C, Smit HA, et al Body mass index trajectory classes and incident asthma in childhood: results from 8 European Birth Cohorts—a Global Allergy and Asthma European Network initiative. J Allergy Clin Immunol. 2013;131(6):1528–36. Epub 2013/02/14. 10.1016/j.jaci.2013.01.001 . 23403049

[13] Garden F, Simpson JM, Mellis CM, Marks GB. Change in the manifestations of asthma and asthma-related traits in childhood: a latent transition analysis. Eur Respir J. 2016;47:362–65. 10.1183/13993003.02011-2015 26493805

[14] Herr M, Just J, Nikasinovic L, Foucault C, Le Marec AM, Giordanella JP, et al Risk factors and characteristics of respiratory and allergic phenotypes in early childhood. J Allergy Clin Immunol. 2012;130(2):389–96 e4. Epub 2012/08/01. 10.1016/j.jaci.2012.05.054 . 22846748

[15] Lazic N, Roberts G, Custovic A, Belgrave D, Bishop C, Winn J, et al Multiple atopy phenotypes and their associations with asthma: similar findings from two birth cohorts. Allergy. 2013;68:764–70. 10.1111/all.12134 23621120

[16] Spycher BD, Silverman M, Brooke AM, Minder CE, Kuehni CE. Distinguishing phenotypes of childhood wheeze and cough using latent class analysis. Eur Respir J. 2008;31(5):974–81. Epub 2008/01/25. 10.1183/09031936.00153507 . 18216047

[17] Spycher BD, Silverman M, Pescatore AM, Beardsmore CS, Kuehni CE. Comparison of phenotypes of childhood wheeze and cough in 2 independent cohorts. J Allergy Clin Immunol. 2013;132(5):1058–67. Epub 2013/10/01. 10.1016/j.jaci.2013.08.002 . 24075230

[18] Anckarsäter H, Lundström S, Kollberg L, Kerekes N, Palm C, Carlström E, et al The Child and Adolescent Twin Study in Sweden (CATSS). Twin Res Hum Gen. 2012;14(06):495–508. 10.1375/twin.14.6.495 22506305

[19] Almqvist C, Ortqvist AK, Ullemar V, Lundholm C, Lichtenstein P, Magnusson PK. Cohort Profile: Swedish Twin Study on Prediction and Prevention of Asthma (STOPPA). Twin Res Hum Genet. 2015;18(3):273–80. 10.1017/thg.2015.17 . 25900604

[20] Asher MI, Keil U, Anderson HR, Beasley R, Crane J, Martinez F, et al International study of asthma and allergies in childhood (ISAAC): rationale and methods. Eur Resp J. 1995;8(3):483–91. 10.1183/09031936.95.08030483 7789502

[21] Ludvigsson JF, Almqvist C, Bonamy AE, Ljung R, Michaelsson K, Neovius M, et al Registers of the Swedish total population and their use in medical research. Eur J Epidemiol. 2016;31:125–36. 10.1007/s10654-016-0117-y . 26769609

[22] Lindemalm C, Nordlund B, Ortqvist AK, Lundholm C, van Hage M, Gong T, et al Associations Between Asthma and Sensitization to Pet or Pollen Allergens in Young Swedish Twins—The STOPPA Study. Twin Res Hum Genet. 2017;20(5):380–8. 10.1017/thg.2017.48 . 28975873

[23] Quanjer PH, Stanojevic S, Cole TJ, Baur X, Hall GL, Culver BH, et al Multi-ethnic reference values for spirometry for the 3-95-yr age range: the global lung function 2012 equations. Eur Respir J. 2012;40(6):1324–43. 10.1183/09031936.00080312 . 22743675 PMC3786581

[24] Martinez F, Morgan WJ, Wright A, Holberg CJ, Taussig LM. Diminised lung function as a predisposing factor for wheezing respiratory illness in infants. NEJM. 1988;319:1112–7. 10.1056/NEJM198810273191702 3173442

[25] Stern DA, Morgan WJ, Wright AL, Guerra S, Martinez FD. Poor airway function in early infancy and lung function by age 22 years: a non-selective longitudinal cohort study. Lancet. 2007;370(9589):758–64. 10.1016/S0140-6736(07)61379-8 17765525 PMC2831283

[26] Owens L, Laing IA, Zhang G, Le Souef PN. Infant lung function predicts asthma persistence and remission in young adults. Respirology. 2016;22:2. 10.1111/resp.12901 27637998

[27] Raza A, Kurukulaaratchy RJ, Grundy JD, Clayton CB, Mitchell FA, Roberts G, et al What does adolescent undiagnosed wheeze represent? Findings from the Isle of Wight Cohort. Eur Respir J. 2012;40(3):580–8. Epub 2012/01/24. 10.1183/09031936.00085111 . 22267759

[28] Ullemar V, Lundholm C, Almqvist C. Twins’ risk of childhood asthma mediated by gestational age and birthweight. Clin Exp Allergy. 2015;45(8):1328–36. 10.1111/cea.12547 . 25845700

