# Supplementary material for: A modern approach to identifying and characterizing child asthma and wheeze phenotypes based on clinical data
Source: PLoS One. 2019 Dec 30;14(12):e0227091. doi: 10.1371/journal.pone.0227091 (PMC6936778; doi:10.1371/journal.pone.0227091)
Supplement: S1 Table — (DOCX) [file pone.0227091.s001.docx]

**Supplementary Material -** *A modern approach to identifying and characterizing child asthma and wheeze phenotypes based on clinical data.*

**B Brew, F Chiesa, C Lundholm, A Örtqvist, C Almqvist**

**S1 Table. Questions from CATSS and STOPPA questionnaires**

| **Swedish (original)** | **English** |
| --- | --- |
| CATSS |  |
| Har eller har <?> haft asthma? | Has s/he ever had asthma? |
| Hur gammal var <?> när han\hon fick ${astman} ${pipande andning} ${andnöd} för första gången? Räkna hela år, t ex var personen 5 månader gammal räknas det som 0 år. | How old was s/he when s/he had asthma/ wheeze/ breathlessness for the first time? |
| Hur gammal var <?> när han\hon senast hade symptom av ${astman} ${pipande andning} ${andnöd} ? | How old was s/he the last time s/he had a symptom of asthma, wheeze, breathlessness? |
| STOPPA |  |
| Har ditt barn någonsin haft pipande eller väsande andning? | Has your child ever had wheezing or whistle breathing? |
| Har ditt barn haft pip, eller har det väst i bröstet vid något tillfälle, under de senaste 12 månaderna? | Has your child had wheeze or whistle in the chest in the last 12 months? |
| Hur många gånger har ditt barn haft pipande eller väsande andning under de senaste 12 månaderna? | How many times has your child had wheeze or whistle breathing in the last 12 months? |
| Vid förkylningar (Vid vilka tillfällen har ditt barn pipande eller väsande andning? ) | On what occasions does your child have wheeze or whistle breathing –colds? |
| Har ditt barn astma? | Does your child have asthma? |
| Har en läkare ställt diagnosen astma? | Has the asthma been diagnosed by a doctor? |
| År gammal (Vid vilken ålder fick ditt barn diagnosen astma? ) | What age was your child when they were diagnosed with asthma? |
| Under de senaste 12 månaderna har astman (pipade eller väsande andningen) begränsat barnets tal till bara ett eller två ord i taget mellan andetagen? | Over the past 12 months has asthma (wheezing or wheezing) limited the child's speech to just one or two words at a time between breaths? |
| Hur ofta har ditt barn haft andningssvårigheter på grund av astma under de senaste 12 månaderna? | How often has your child had breathing difficulties due to asthma in the last 12 months? |
| Hur många gånger har ditt barn blivit väckt av sin astma under de senaste 12 månaderna? | How many times has your child been woken by asthma in the last 12 months? |
| Hur mycket har ditt barn störts av sin astma i sina dagliga aktiviteter under de senaste 12 månaderna? | How many times has asthma interfered with your child’s daily activities in the last 12 months? |
| Har barnet behövt söka hjälp akut på akutmottagning eller vårdcentral på grund av astma de senaste 12 månaderna? | Has the child had to seek emergency help at an emergency room or medical center due to asthma in the past 12 months? |
| Har ditt barn blivit inlagd på sjukhus för astmabesvär under de senaste 12 månaderna? | Has your child been hospitalised for asthma in the last 12 months? |
| Snarkar ditt barn för närvarande? | Does your child snore? |
| Har mamman astma? | Does the mother have asthma? |
| Röker mamman(cigaretter/cigariller/cigarr/pipa)? | Does the mother smoke? |
| Röker pappan (cigaretter/cigariller/cigarr/pipa)? | Does the father smoke? |
| Har pappan astma? | Does the father have asthma? |
| Finns det, eller har det funnits pälsdjur i tvillingarnas hem? Hund (0-4 års ålder) | Is there, or has there been fur animals in the twins' homes? Dog (0-4 years) |
| Ammades den förstfödde tvillingen? | Was the twin breastfed? |
| Har eller har barnet haft eksem? | Does s/he have eczema? |
| Har eller har barnet haft hösnuva? | Does s/he have hayfever? |
| Tar ditt barn för närvarande någon astmamedicin (inklusive inhalatorer, sprejer och tabletter)? | Is your child currently taking any asthma medication (including inhalers, sprays and tablets)? |
| Har ditt barn tagit snabbverkande luftrörsvidgande medicin (t.ex. Bricanyl, Ventoline, Buventol eller Airomir) på grund av astmabesvär mer än två gånger den senaste veckan? | Has your child been taking fast-acting bronchodilator medication (eg Bricanyl, Ventoline, Buventol or Airomir) due to asthma problems more than twice in the past week? |
| Har ditt barn under de senaste 12 månaderna använt följande mediciner? Markera alla aktuella | Has your child used the following medicines in the last 12 months? Mark all current ones. |
| 1. Kortison för inandning (t.ex. Pulmicort, Flutide, Becotide, Asmanex, Beclomet ) | 1. Cortisone for inhalation (eg Pulmicort, Flutide, Becotide, Asmanex, Beclomet) |
| 1. Långverkande luftrörsvidgande (t.ex. Serevent, Oxis) | 1. Long-acting bronchodilation (eg, Serevent, Oxis) |
| 1. En kombination av långverkande luftrörsvidgande och kortison (t.ex. Seretide, Symbicort) | c. A combination of long-acting bronchodilation and cortisone (eg Seretide, Symbicort) |
| 1. Singulair (tabletter) | d. Singulair (tablet) |
| 1. Kortisontabletter (t.ex. Prednisolon, Betapred) | e. Cortisone tablet (eg Prednisolone, Betapred) |
