# Supplementary material for: A modern approach to identifying and characterizing child asthma and wheeze phenotypes based on clinical data
Source: PLoS One. 2019 Dec 30;14(12):e0227091. doi: 10.1371/journal.pone.0227091 (PMC6936778; doi:10.1371/journal.pone.0227091)
Supplement: S2 Table — AIC = Aikake Information Criteria, BIC = Bayesian Information Criteria. (DOCX) [file pone.0227091.s002.docx]

**Supplementary Material -** *A modern approach to identifying and characterizing child asthma and wheeze phenotypes based on clinical data.*

**B Brew, F Chiesa, C Lundholm, A Örtqvist, C Almqvist**

**S2 Table. Comparison of 4, 5 and 6 latent class models. AIC= Aikake Information Criteria, BIC = Bayesian Information Criteria**

| Number of classes | BIC | AIC | Entropy Index | Lo-Mendell- Rubin |
| --- | --- | --- | --- | --- |
| **4** | 5351.57 | 5004.87 | 1.00 | VUONG-LO-MENDELL-RUBIN LIKELIHOOD RATIO TEST FOR 3 (H0) VERSUS 4 CLASSES  H0 Loglikelihood Value -2601.862  2 Times the Loglikelihood Difference 348.857  Difference in the Number of Parameters 19  Mean -4.295  Standard Deviation 22.560  P-Value 0.0000  LO-MENDELL-RUBIN ADJUSTED LRT TEST  Value 346.106  P-Value 0.0000 |
| **5** | 5307.05 | 4872.51 | 0.93 | VUONG-LO-MENDELL-RUBIN LIKELIHOOD RATIO TEST FOR 4 (H0) VERSUS 5 CLASSES  H0 Loglikelihood Value -2427.434  2 Times the Loglikelihood Difference 170.359  Difference in the Number of Parameters 20  Mean 6.277  Standard Deviation 5.399  P-Value 0.0000  LO-MENDELL-RUBIN ADJUSTED LRT TEST  Value 169.083  P-Value 0.0000 |
| **6** | 5400.023 | 4877.654 | 0.92 | VUONG-LO-MENDELL-RUBIN LIKELIHOOD RATIO TEST FOR 5 (H0) VERSUS 6 CLASSES  H0 Loglikelihood Value -2342.254  2 Times the Loglikelihood Difference 32.854  Difference in the Number of Parameters 19  Mean 25.030  Standard Deviation 21.963  P-Value 0.2466  LO-MENDELL-RUBIN ADJUSTED LRT TEST  Value 32.595  P-Value 0.2498 |
